# The durability of different anchoring points and anchoring types in the left ventricle in an ex vivo porcine model

**DOI:** 10.1111/cpf.70099

**Published:** 2026-10-15

**Authors:** Henrik Bjursten, Per Wierup, Sasha Koul, Matthias Götberg, Magnus Dencker

**Affiliations:** ^1^ Department of Cardiothoracic Surgery Lund University, Skåne University Hospital Lund Sweden; ^2^ Department of Cardiothoracic Surgery Cleveland Clinic Cleveland Ohio USA; ^3^ Department of Clinical Sciences Lund Cardiology, Lund University, Skåne University Hospital Lund Sweden; ^4^ Department of Medical Imaging and Physiology Skåne University Hospital Malmö Sweden; ^5^ Department of Translational Medicine Lund University Lund Sweden

**Keywords:** anchoring, durability, ex vivo, Mitral valve, strain

## Abstract

**Objective:**

Surgical mitral valve repair, including anchoring in different parts of the mitral apparatus, is the standard of care for severe mitral regurgitation. Various percutaneous approaches have been developed and are currently under development, but are struggling with durable results. This study investigates the durability of different anatomic anchoring points and anchoring types in the left ventricle.

**Material and Methods:**

Anchoring was tested with PTFE sutures or a 5 mm helix at three locations: the head of the papillary muscle, the lateral wall of the ventricle, and the apex of the ventricle. An accelerated life test was performed with 300 cycles at 3 N for each anchoring. Durability was assessed by measuring compliance (strain in mm until 3 N is reached), migration (change in strain from the first to the last cycle), and the occurrence of dehiscence.

**Results:**

The lowest numerical strain/compliance in the first cycle was observed at the head of the papillary muscle anchoring (both surgical and helix). Migration of strain limits over 300 cycles was seen for all locations. The lowest migration was numerically for helix in the papillary muscle.

**Conclusion:**

PTFE in the head of the papillary muscle is standard practice in mitral valve repair and was used as a reference in the present study. The anchoring that yielded the most similar results was a helix in the papillary muscle, whereas other anchoring points showed greater compliance and/or migration.

## 1 INTRODUCTION

The mitral apparatus is a very complex structure that includes the annulus, mitral leaflets, chordae, papillary muscles, and the left ventricle, all of which play important roles in the opening and closing of the mitral valve (Sacks et al., 2019; Silbiger, 2012). In a diseased regurgitant valve, one or more of these structures have undergone conformational changes. Mitral valve regurgitation is normally divided into functional mitral regurgitation (FMR) or degenerative mitral regurgitation, where the former is primarily a disease of the left ventricle and annulus and the latter a disease of the chordae and leaflets. Traditionally, open‐heart surgery has been offered to patients with severe mitral disease, and normally, a good surgical repair offers excellent long‐term results (David et al., 2019). However, the path for this surgical success has evolved during a period of 50 years (Fedak et al., 2008; Bortolotti et al., 2012).

In recent years, transcatheter treatment of mitral valve disease has started to evolve, in the form of either transcatheter mitral valve replacement (TMWR) or transcatheter mitral valve repair (TMWr). Well over 30 technologies are at various stages of development (Stone et al., 2018), with the most successful and most mature being transcatheter edge‐to‐edge repair (TEER) for FMR (Stone et al., 2018, 2023).

One challenge many devices face is performing a repair that respects the complexity of the mitral apparatus, delivers good long‐term results, and maintains the option for a later surgical repair. Given the intricacy of the mitral apparatus, many issues remain unresolved, including achieving adequate anchoring in the left ventricle for a TMWr device. In surgery, this has been addressed by emulating nature through chordal reconstructions using PTFE chords anchored at the fibrous tips of the papillary muscles, which yield good long‐term results (David et al., 2019). Different anchoring points have been tested for TMWr devices, and most have not shown acceptable long‐term outcomes (Rogers and Bolling, 2021). It is therefore important to explore the effect of different anchoring locations and types on durability. This study aims to evaluate the durability of three different anchoring points, both for surgical sutures and helices, in an explanted heart model.

## 2 METHODS

### 2.1 Study design

An experimental setup was designed to test the durability of different left ventricular anchoring points. As a myocardial model, we used explanted porcine hearts from adult pigs. The heart was cleaved below the mitral annulus and opened by an incision from the annular plane almost all the way to the apex. Anchoring was performed in three locations (head of papillary muscle, lateral wall of ventricle, and apex of ventricle). In each location, we anchored with both CV4 PTFE sutures (W. L. Gore & Associates, Newark, DE) or a 5 mm helix (Figure 1). Surgical placement was performed in the same manner as when performing a mitral chordae repair (Pfannmüller et al., 2014). For helical anchoring, we used the EndoCap technology to grasp the papillary head and secure the helix (Bjursten et al., 2019). The helices were placed using an EndoCap prototype, but negative‐pressure grasping was used only for the papillary muscle head. The device has been developed for grasping and holding beating heart tissue using controlled negative pressure, enabling precise placement of helices in a beating organ. A Zwick/Roell Z2.5 (Zwick Roell AG, Ulm, Germany) with a 10 Newton load cell was used to exert the strain forces (Central illustration 1).

**FIGURE 1:**
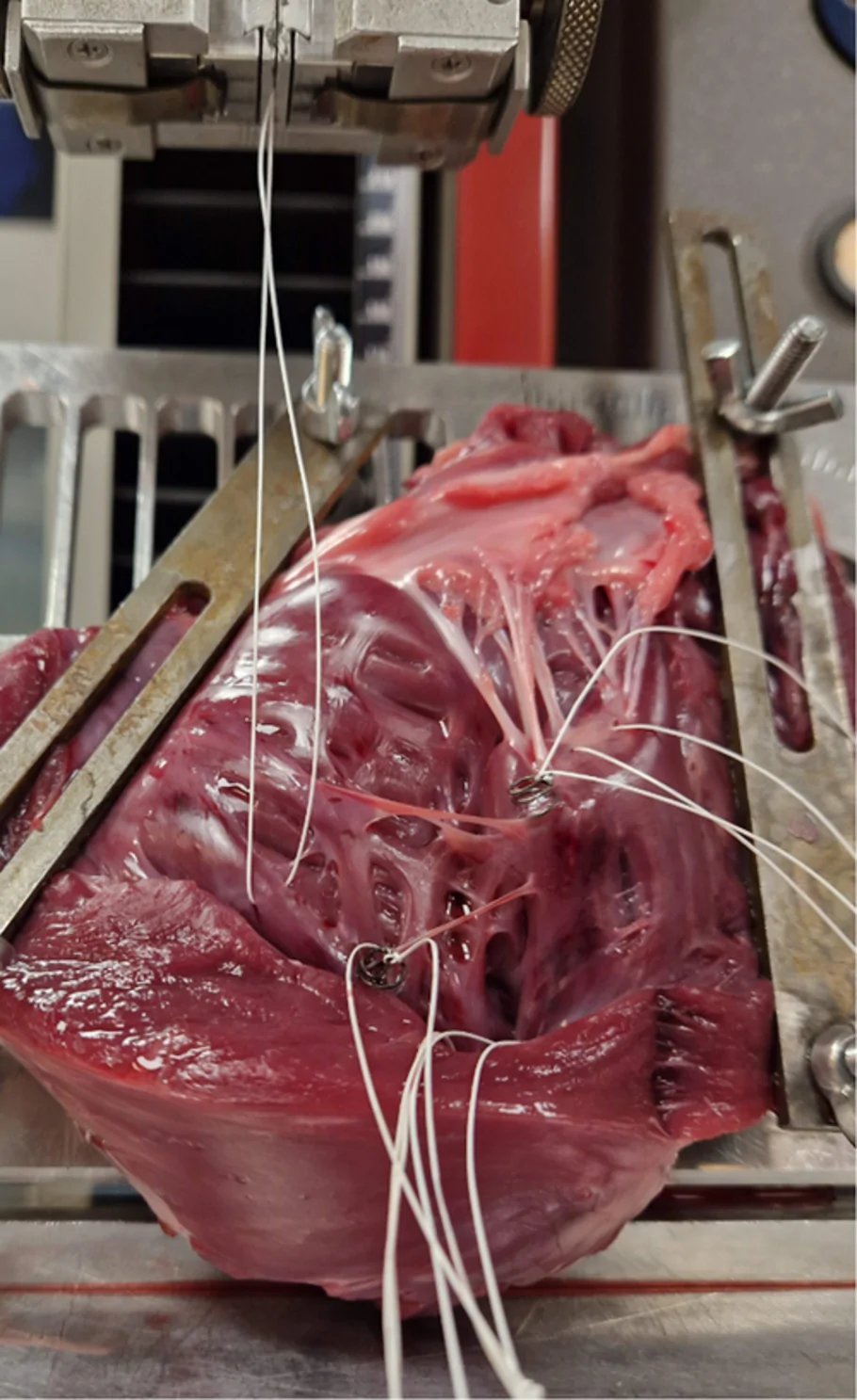
Opened left ventricle of a heart with different anchoring points mounted in the strain testing device. a=surgical papillary, b=helix papillary, c=surgical lateral wall, d=helix lateral wall, e=surgical apex, f=helix apex.

**Central illustration 1:**
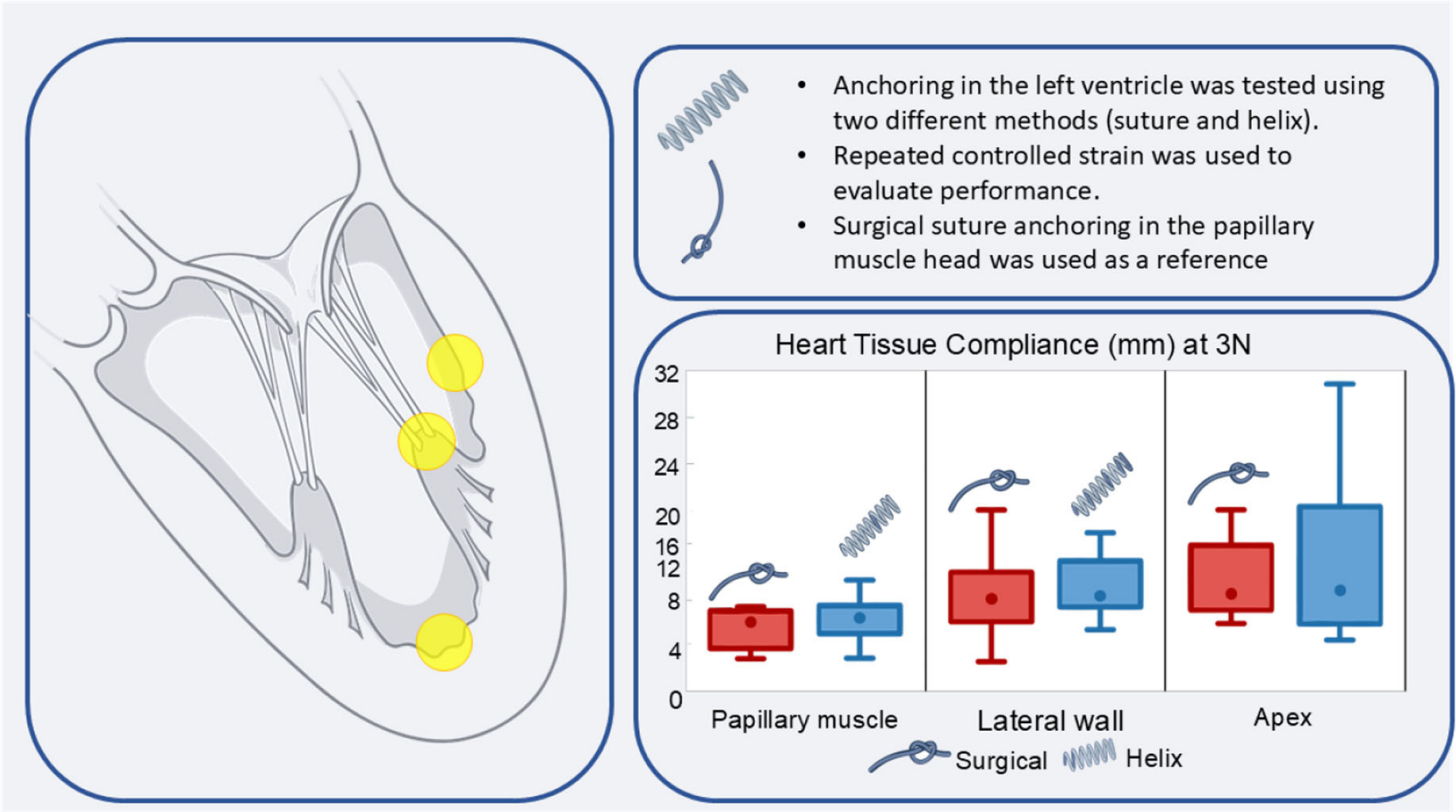
Testing of different anchoring points and modalities in the left ventricle.

### 2.2 Study protocol

For this study, we employed accelerated life testing, as first described by Nelson in 1980 (Tribouilloy et al., 1999), in which a supranormal force is applied to shorten the test procedure.

At first, one heart was used for testing increasing loads on the anchoring points. Strain testing started at 0.2 N and increased to 0.4 N, 0.8 N, 1.6 N, 3.2 N, and 6.4 N, with 50 pulls per cycle. The data from this cycle were used to determine the setup for the second part of the study. In this part of the study, we used 8 explanted hearts and applied 3.0 N as a predetermined force for 300 cycles (approximately 30 min) at each location. The order across the different locations tested was cycled so that each location received a new order for every heart. Strain in mm was recorded at cycles 1, 5, 10, 15, 20, 25, 50, 75, 100, 125, 150, 175, 200, 225, 250, 275, and 300. If there was a dehiscence of an anchoring point, the pull cycle in which it happened was recorded. For the study, we created variables for strain from 0.01 N (load start) to 0.6 N and from 0.01 N to 3 N. From this measurement, we defined two variables.

### 2.3 Outcome variables

*Compliance* was defined as the movement length, measured in mm, from 0.1 N to 0.6 N or to 3 N for the first cycle.

*Migration* was defined as the difference in maximal movement between cycles 1 and 300 at strain 0.6 N and 3 N, respectively.

### 2.4 Statistical analysis

Numbers are mostly presented as medians with interquartile ranges, but in some instances, the mean with standard deviation is also presented. Testing was performed using the Mann–Whitney *U* test. Surgical papillary was used as a reference for testing, as this is standard practice in mitral valve surgery. Statistical analysis was performed with Statistica ver. 14 (TIBCO Software).

## 3 RESULTS

Testing was successful for all eight tested hearts. There was dehiscence of the anchoring points in 6 (of 8) hearts for surgical anchoring in the lateral wall, 1 (of 8) in the apex, and none in the papillary muscle. There was no dehiscence for the helical anchoring. The typical strain testing cycle at cycle 100 is shown in the video supplements. Mean test results for each location, together with failure points, are presented in Figure 2, 3.

**FIGURE 2:**
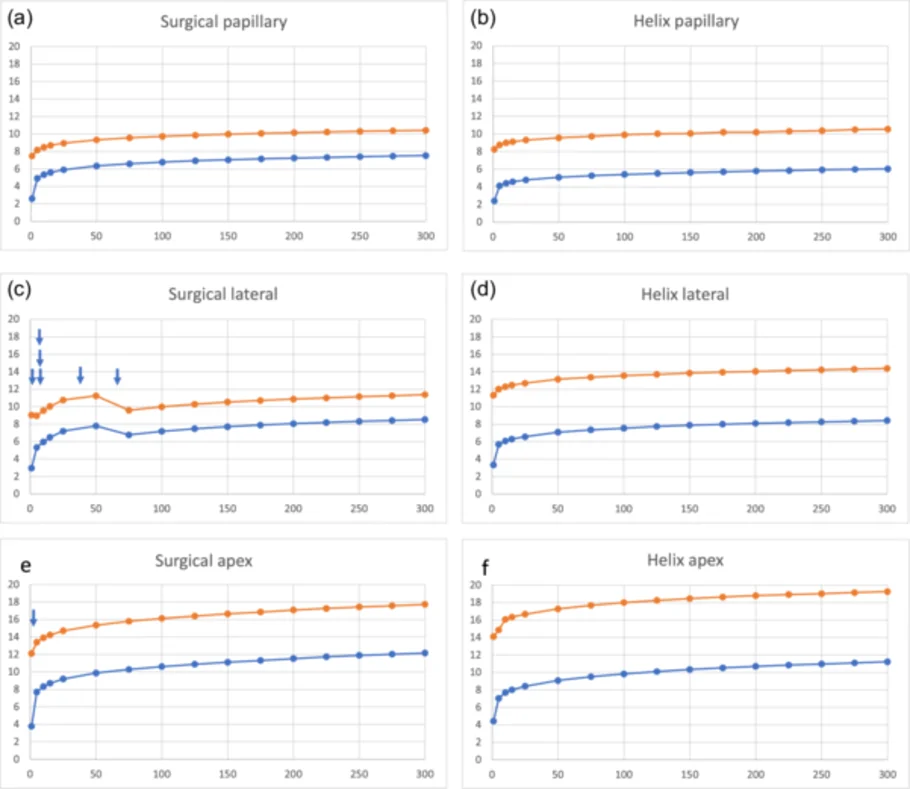
Strain recording from cycle 1–300 for 0.6 N (blue line) and 3 N (red line) for each location and anchoring type. (a) Surgical papillary, (b) Helix papillary, (c) Surgical lateral, (d) Helix lateral, (e) Surgical apex, and (f) Helix apex. Cycle number on the *X*‐axis, and strain in mm till predetermined load on the *Y*‐axis. Arrows indicate one dehiscence of anchoring.

The lowest numerical compliance for the first cycle was seen for papillary muscle anchoring (both surgical and helix). Compared with surgical papillary anchoring, there was no difference in compliance strain at 0.6 N. At 3 N, surgical apex (10.7 mm [9.4–15.2], *p* = 0.006), helix lateral (10.5 mm [9.6‐13.4], *p* = 0.005), and helix apex (10.8 mm [8.1–18.4], *p* = 0.04) were all higher than surgical papillary (8.1 mm [5.7–9.0], Table 1 and Figure 3).

**FIGURE 3:**
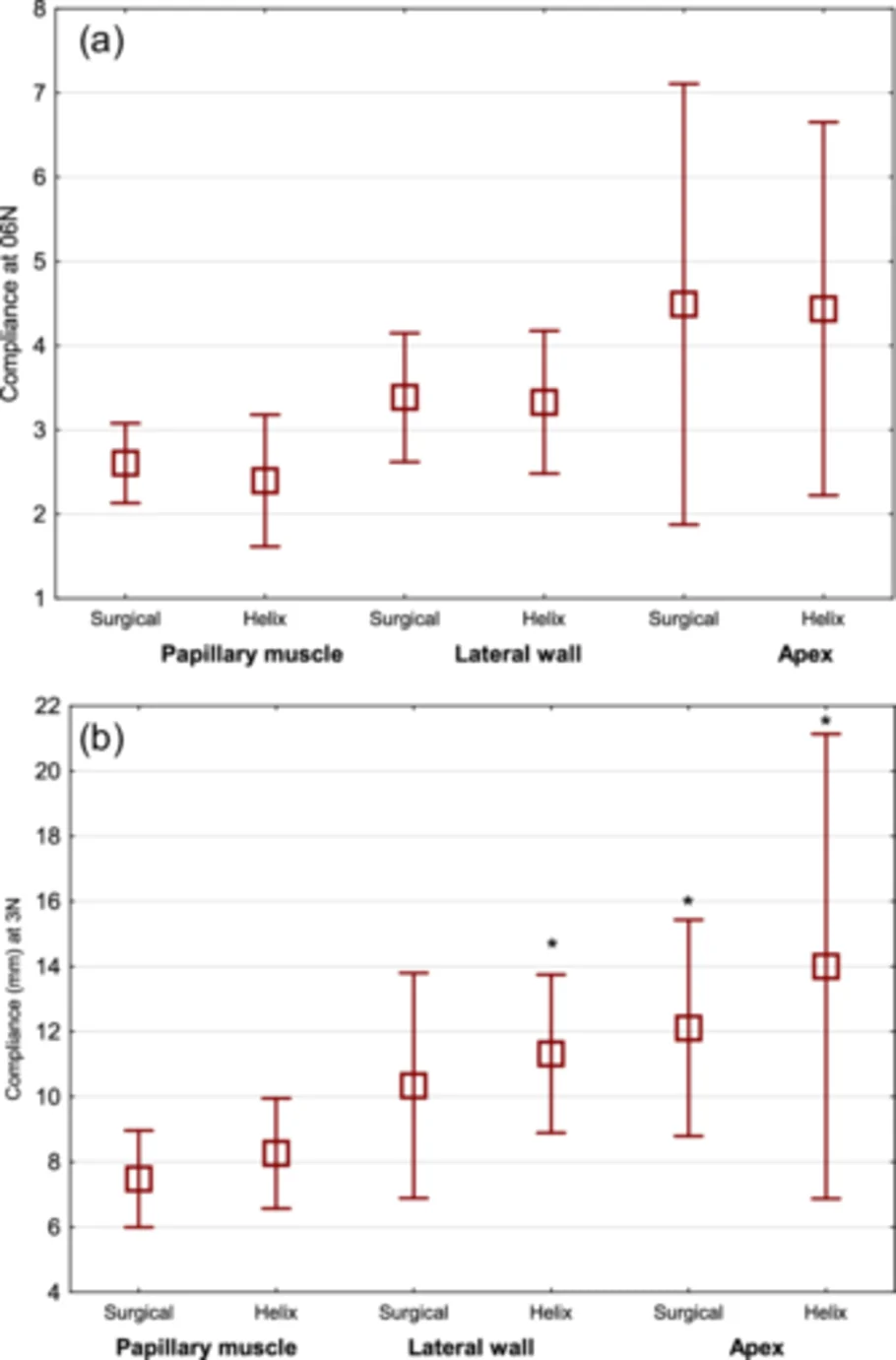
Compliance (strain in mm) from load start (0.01 N) to load 0.6 N (a ‐ upper) and load 3 N (b ‐ lower) for the first cycle. Data represented as mean with 95% confidence interval.

**TABLE 1:** Compliance (strain in mm) for the first cycle from load start (0.01 N) to 0.6 N and 3 N, respectively. Testing with the Mann–Whitney *U*‐test against the strain for surgical papillary.

	Mean ± sd	Median (IQR)	*p*‐value
**Strain at 0.6 N**			
Surgical ‐ Papillary	2.6 ± 0.6	2.8 (2.1–3.0)	ref
Surgical ‐ Lateral	3.0 ± 1.4	3.3 (2.5–3.6)	0.141
Surgical ‐ Apex	4.5 ± 2.8	3.0 (2.4–8.0)	0.183
Helix ‐ Papillary	2.4 ± 0.9	2.6 (1.6‐3.0)	0.674
Helix‐ Lateral	3.3 ± 1.0	3.0 (2.6–4.0)	0.207
Helix ‐ Apex	4.4 ± 2.7	3.1 (2.4–6.8)	0.248
**Strain at 3 N**			
Surgical ‐ Papillary	7.5 ± 1.8	8.1 (5.7–9.0)	ref
Surgical ‐ Lateral	9.0 ± 5.0	9.7 (6.4–11.9)	0.318
Surgical ‐ Apex	12.1 ± 3.6	10.7 (9.4–15.2)	0.006
Helix ‐ Papillary	8.3 ± 2.0	8.5 (7.1–9.2)	0.495
Helix‐ Lateral	11.3 ± 2.9	10.5 (9.6–13.4)	0.005
Helix ‐ Apex	14.0 ± 8.5	10.8 (8.1–18.4)	0.040

Strain limit migration was observed at all locations (Figure 1). Numerically, the lowest migration was for helix in the papillary muscle, both at 0.6 N and 3 N. At 0.6 N, only the surgical apex (8.9 mm [6.9–9.1], *p* = 0.006) had significantly higher migration than the surgical papillary (5.1 mm [3.5‐6.3], Table 2). At 3 N, both surgical apex (5.4 mm [3.5–7.7], *p* = 0.009) and helix apical (4.7 mm [3.8–7.3], *p* = 0.03) were higher than surgical papillary (2.7 mm [2.5–3.6], Table 2 and Figure 4).

**TABLE 2:** Migration (strain change/delta) from the first cycle to cycle 300 for 0.6 N and 3 N load, respectively. Testing with the Mann–Whitney *U*‐test against the strain for surgical papillary.

	Mean ± sd	Median (IQR)	*p*‐value
Δ‐Strain at 0.6 N			
Surgical ‐ Papillary	4.9 ± 1.7	5.1 (3.5–6.3)	ref
Surgical ‐ Lateral	5.8 ± 0.4	5.8 (5.5–6.1)	0.513
Surgical ‐ Apex	8.7 ± 2.7	8.9 (6.9–9.1)	0.006
Helix ‐ Papillary	3.6 ± 0.8	3.6 (3.2–4.4)	0.103
Helix‐ Lateral	5.1 ± 1.2	5.2 (3.9–6.0)	0.793
Helix ‐ Apex	6.8 ± 3.8	4.8 (4.2–9.3)	0.495
Δ‐Strain at 3 N			
Surgical ‐ Papillary	2.9 ± 1.1	2.7 (2.5–3.6)	ref
Surgical ‐ Lateral	5.0 ± 0.3	5.0 (4.8–5.2)	0.050
Surgical ‐ Apex	5.6 ± 2.0	5.4 (3.5–7.7)	0.009
Helix ‐ Papillary	2.3 ± 0.9	2.2 (1.7–2.5)	0.104
Helix‐ Lateral	3.1 ± 0.8	3.2 (2.4–3.7)	0.563
Helix ‐ Apex	5.4 ± 2.4	4.7 (3.8–7.3)	0.031

**FIGURE 4:**
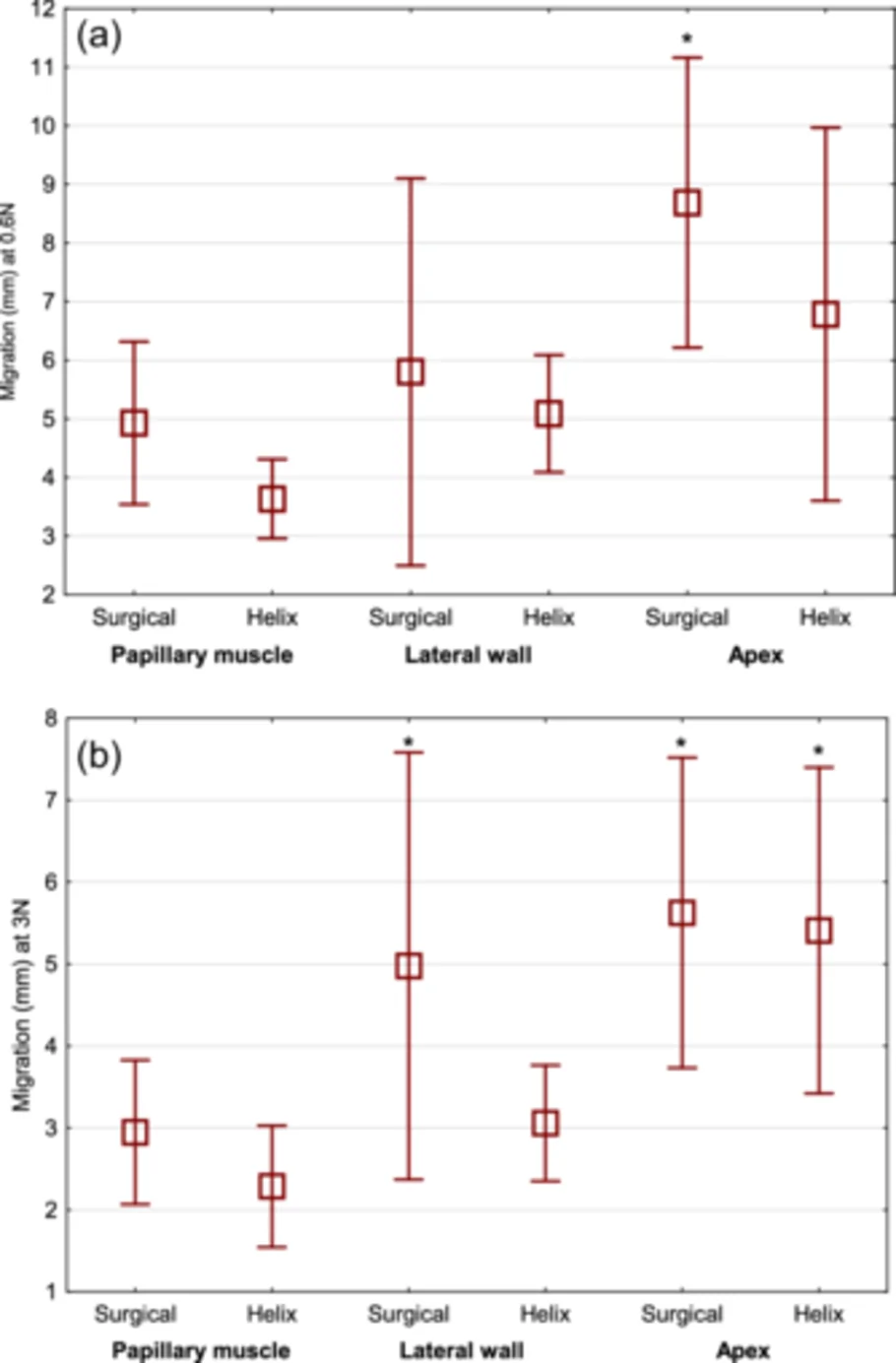
Migration (strain migration) from cycle 1 to cycle 300 at load 0.6 N (a ‐ upper) and load 3 N (b ‐ lower). Data represented as mean with 95% confidence interval.

## 4 DISCUSSION

The current study was undertaken to learn more about the durability of tether anchoring at different locations and using different techniques in the left ventricle for mitral chordae repair. As placing a PTFE suture in the head of the papillary muscle is standard practice in mitral valve repair, this was used as a reference and comparison for the other anchoring methods. The anchoring that yielded the most similar results was a helix in the papillary muscle, while the other showed greater compliance and/or migration. The least favourable anchoring sutures in the lateral wall, where 6 of 8 tests failed due to dehiscence. Mitral valve repair is the gold standard for mitral regurgitation, and when performed at optimal timing, long‐term survival is comparable to that of the background population (Kang et al., 2009; Montant et al., 2009; Tribouilloy et al., 1999). Initially, leaflet resection techniques were used based upon the pioneering work of Prof Carpentier (Carpentier, 1983). However, given that the primary goal of mitral valve repair is to restore a smooth and large surface of coaptation and that this zone of coaptation consists of leaflet tissue, it is logically appealing to preserve it. Hence, non‐resectional techniques have been developed and are now the dominating techniques. Perier et al. introduced the concept of “respect rather than resect” published in 2008 (Perier et al., 2008). The long‐term durability of this technique has been published extensively (David et al., 2019; Holubec et al., 2013). The common denominator for these techniques is the placement of neochords using PTFE sutures. Surgical experience learned that they should always be anchored in the fibrous tips of the papillary muscles. There are increased demands for less invasive techniques to achieve mitral valve repair, and ultimately, percutaneous solutions are desirable. The TEER technique is established but requires careful judgement to avoid functional mitral valve stenosis. Chordal reconstruction, on the other hand, preserves a large mitral opening while at the same time addressing each segmental prolapse. The key is, however, to have the PTFE sutures adequately anchored and hence this study.

One conclusion of this study is that myocardial tissue quality differs across locations. For instance, the myocardium in the free wall of the ventricle is frail, as 6 of 8 sutures here cut through the myocardium and detached early in the testing. This happened in 1 of 8 sutures in the apex. This might not be that surprising, as most of the ventricular wall and apex consist of mostly muscle fibres and little collagen (Miles et al., 2020). Whereas the head of the papillary head, where there is a transition between myocardium and chordae, is very collagen dense (Hutchison and Rea, 2015), and no dehiscence was observed. In addition, myocardial fibre direction may play a role when anchoring in myocardial tissue, and fibres are directed in the direction of the heart's contraction (Kocica et al., 2006), which might not be optimal for strain resistance in other places than the papillary muscle. A study by Axel found that the base of the papillary can serve as a shock absorber (Axel, 2004), further strengthening the case for papillary muscle anchoring.

All helical anchoring points endured the accelerated strain testing without dehiscence. As helices are the predominant way for ventricular anchoring in TMWr, this is an interesting observation. However, a few devices that have tested helical anchoring in the apex have failed to show long‐term durability (Rogers and Bolling, 2021). This leads to the question of whether the explanation for dehiscence in humans can be found in this study. When comparing helical anchoring in the papillary muscle and in the lateral wall or apex, we made two observations. First, the strain testing for the first cycle showed a longer distance to the predetermined strain than for papillary muscle anchoring, and the strain's standard deviation was also larger than for papillary muscle anchoring. Second, migration over time was greater for apical anchoring than for papillary muscle anchoring. In this case, it also has a bigger standard deviation. This finding infers that the tethering of the new chordae in a TMWr will have a large variation and increase over time. Consequently, a flail might develop over time, which also increases the load on the new chordae. Also, testing sutures in the lateral wall and apex in this series clearly shows that these locations are unsuitable for anchoring. Other factors determine how well a helical anchor will hold over time. First, the design of the helix in terms of diameter, depth and gap between the loops. Second is, of course, how well the helix is anchored in the myocardium, and achieving controlled anchoring in a beating heart with a catheter controlled from the groin is a challenge. This might pose an extra challenge when trying to anchor in the apex or the lateral wall, as both areas have many trabeculae. The EndoCap technology was designed not only to anchor in the papillary muscle but also to achieve controlled, repeatable anchoring at a predetermined depth.

This model, which uses explanted hearts, has many limitations for extrapolating its findings to a beating human heart. First of all, it is non‐beating, dead tissue from another species. We tried to replicate the strain exerted with every heartbeat using a strain‐testing device, but the normal closing of the mitral valve is far more complex. A new chordae should withstand millions of cycles, which is impossible to replicate in the current model. In an effort to compensate for this, we used an accelerated life‐testing model with forces 5–10× physiological (Jimenez et al., 2005; Sacks et al., 2019).

## 5 CONCLUSION

While this ex vivo study has limitations, it demonstrates that anchoring a new chordae to the papillary head during surgery is the optimal location, consistent with clinical experience. The study also suggests that, to achieve similar dynamics with a helical anchor, it must be placed in the papillary head.

## AUTHOR CONTRIBUTIONS

**Henrik Bjursten:** study design, study execution, analysis, writing. **Per Wierup:** study design, manuscript preparation. **Sasha Koul:** manuscript preparation. **Matthias Götberg:** manuscript preparation. **Magnus Dencker:** study design, manuscript preparation.

## CONFLICT OF INTEREST STATEMENT

As the EndoCap is an invention from Lund University, HB, PW, MG, and MD have vested interests in the technology.

## Data Availability

The data that support the findings of this study are available on request from the corresponding author. The data are not publicly available due to privacy or ethical restrictions.
